# The computational implementation of a platform of relative identity-by-descent scores algorithm for introgressive mapping

**DOI:** 10.3389/fgene.2022.1028662

**Published:** 2023-01-24

**Authors:** Bo Cui, Zhongxu Guo, Hongbo Cao, Mario Calus, Qianqian Zhang

**Affiliations:** ^1^ School of Chemistry and Biological Engineering, University of Science and Technology, Beijing, China; ^2^ College of Water Resources and Civil Engineering, China Agricultural University, Beijing, China; ^3^ Institute of Biotechnology, Beijing Academy of Agricultural and Forestry Sciences, Beijing, China; ^4^ Department of Animal Science, Animal Breeding and Genetics Group, Wageningen University, Wageningen, Netherlands

**Keywords:** adaptive introgression, hybridization, identity by descent, gene flow, algorithm, computational platform

## Abstract

With the development of genotyping and sequencing technology, researchers working in the area of conservation genetics are able to obtain the genotypes or even the sequences of a representative sample of individuals from the population. It is of great importance to examine the genomic variants and genes that are highly preferred or pruned during the process of adaptive introgression or long-term hybridization. To the best of our knowledge, we are the first to develop a platform with computational integration of a relative identity-by-descent (rIBD) scores algorithm for introgressive mapping. The rIBD algorithm is designed for mapping the fine-scaled genomic regions under adaptive introgression between the source breeds and the admixed breed. Our rIBD calculation platform provides compact functions including reading input information and uploading of files, rIBD calculation, and presentation of the rIBD scores. We analyzed the simulated data using the rIBD calculation platform and calculated the average IBD score of 0.061 with a standard deviation of 0.124. The rIBD scores generally follow a normal distribution, and a cut-off of 0.432 and −0.310 for both positive and negative rIBD scores is derived to enable the identification of genomic regions showing significant introgression signals from the source breed to the admixed breed. A list of genomic regions with detailed calculated rIBD scores is reported, and all the rIBD scores for each of the considered windows are presented in plots on the rIBD calculation platform. Our rIBD calculation platform provides a user-friendly tool for the calculation of fine-scaled rIBD scores for each of the genomic regions to map possible functional genomic variants due to adaptive introgression or long-term hybridization.

## 1 Introduction

Adaptive introgression in situations with gene flow between different breeds or even species holds high interest in evolutionary genetics, as the genetic basis of this phenomenon is largely unknown when there is gene flow between different breeds, varieties, species, etc. (Voight et al., 2006; Bosse et al., 2014a; Ai et al., 2015). Identifying the genes or genomic regions that are due to introgression will help understand the genetic basis of long-term hybridization and admixture. It is of great importance to identify which genomic regions or genes influence the phenotypic character or traits of hybrids and how these have interacted with each other to form the genetic basis of hybrid breeds (Bosse et al., 2014b; Jagoda et al., 2017; Zhang et al., 2018). The genomic regions or genes identified with an important role in adaptive introgression hold a higher possibility as a causal variant affecting the phenotypes or traits. For example, Tibetans with altitude adaptation are caused by a genetic background traced long back to Denisovan-like DNA (Huerta-Sanchez et al., 2014; Ai et al., 2015). By identifying these important facts, we are able to disentangle the genetic basis of hybridization and the genetic effects of introgression during adaptation for important phenotypes, traits, or even diseases, which has long been an important research topic receiving much attention.

With the development of genotyping and sequencing technology, it is possible to sequence individuals deeply for all nucleotides with no ascertainment bias (Daetwyler et al., 2014; Zhang et al., 2015). This enables detailed examination of single genomes to understand the phenomenon of introgression and hybridization at the population level, which provides a valid basis for the information of mapping the functional genomic variants in individuals (Ai et al., 2015; Chen et al., 2016; Qanbari et al., 2011; vonHoldt et al., 2016). After the hybridization, some of the genomic variants can be favored or pruned out with a high or low frequency in the population across several generations of directional selection (Hedrick, 2013; Bosse et al., 2014a; Ai et al., 2015; Galov et al., 2015; Hartwig et al., 2015). These introgressed genomic variants are highly likely to be functional and play a key role during the process of introgression and hybridization (Bosse et al., 2014b; Hasenkamp et al., 2015; Deschamps et al., 2016; Figueiro et al., 2017). Mapping these genomic variants that have been subjected to adaptive introgression using a valid method provides information about these variants that can be used for further functional validations.

Many studies have identified these functional genomic variants that have been subjected to adaptive introgression (Bosse et al., 2014b; Deschamps et al., 2016; Figueiro et al., 2017; Wu et al., 2018; Zhang et al., 2018). The aim of these studies is to examine the genome-wide signatures of adaptive divergence and introgression in depth and further disentangle the genetic basis of the complex traits formed during this process by the utilization of genomic analysis from phylogeny. Among these studies, Zhang et al. (2018) utilized the relative identity-by-descent (rIBD) algorithm to identify the genomic regions in an admixed Red cattle breed that arose from adaptive introgression from Holstein and Brown Swiss cattle breeds. Figueiro et al. (2017) found complex genomic signatures of introgression using phylogeny, comparative analysis, and demographic reconstructions, and genes involved in craniofacial and limb development were identified.

So far, there are many tools which can calculate the proportion of admixed genomes obtained from ancestral breeds at the level of an individual, such as ADMIXTURE (Alexander et al., 2009). It is also possible to construct the phylogeny and demographic history using tools such as RAxML (Stamatakis, 2014) and PSMC (Li and Durbin, 2011). These tools and methods could examine the demographic history and population structure on the basis of an individual genome. There is, however, no tool which enables to map these genomic variants at a fine scale, that is, up to base-pair resolution. The algorithm of rIBD was previously applied in our studies for adaptive introgression mapping (Zhang et al., 2018). IBD inference is to detect the haplotypes which are inherited from the common ancestor, and the IBD states could reflect the general pattern of demographic history on the population scale (Sticca et al., 2021). The advantage of IBD detection is that it is even possible to trace back to the recent common ancestor of the shared pattern of rare variation, which has been long mysterious for the research scientists in the field. Therefore, IBD detection is of great importance for addressing the unanswered questions in genomics and genetics.

Here, we develop a user-friendly platform with the implementation of the algorithm of rIBD (Bosse et al., 2014b; Zhang et al., 2018) for identifying genomic variants and genomic regions that have been subjected to introgression in a fine-scaled sliding window across the whole genome from an evolutionary perspective. These genomic variants that have been subjected to adaptive introgression are basically identity by descent with the ancestral breed from which it was derived when comparing pair-wise between individual genomes. The objective of this study is to 1) implement the rIBD algorithm with a fine-scaled sliding window, 2) integrate rIBD algorithm in a user-friendly platform so that the users can utilize it as an online tool, and 3) demonstrate the different aspects of the results calculated using rIBD algorithm and different compact modules of the rIBD calculation platform based on a small simulated dataset.

## 2 Materials and methods

We implement the method of rIBD algorithm into the platform in three steps: 1) input and the design of the front interface: files, options, and illustrations; 2) the rIBD calculations: calculation options; and 3) output: files and illustrations.

We first design the front interface with the options of inputting the files. Here, it is designed to have the input information from source breed 1, source breed 2, and the breed with admixture. It is necessary to calculate the basic identity-by-descent (IBD) information first between source breed 1 and the admixed breed and between source breed 2 and the admixed breed based on the genomic marker input information. Our rIBD scores are calculated based on the posterior inferences of the basic IBD states of shared IBD tracts between two individuals. It is also possible to use the sample data as input for a quick rIBD calculation and demonstration. At the moment, our platform only supports rIBD calculations for one admixed breed at a time that is derived from no more than two ancestral breeds. When there are more ancestral breeds to be considered, the user should perform one separate analysis for each possible pair of ancestral breeds.

The next step is to utilize the input information described previously to calculate the rIBD scores using a sliding window across the whole genome. Here, we design the rIBD algorithm for a genome with a sliding window of 10 kbp, that is, the minimum resolution for the possibility to map a gene on a single genome, is selected as the size of the sliding window. In this way, we could reach the highest resolution to scan the genomes with all possible adaptive introgression signals. Using a sliding window of 10 kbp, we make pair-wise comparisons between each admixed individual and all individuals in source breed 1, and all individuals in source breed 2. The proportion of genomic regions which are calculated as significant IBD is outputted between source breed 1 and the breed with admixture and between source breed 2 and the breed with admixture. A relative IBD (rIBD) score is finally calculated when considering source breed 1, source breed 2, and the breed with admixture in the following format:

$${IBD}_{S1}-{IBD}_{S2},$$ (1)

where IBD_S1_ refers to the proportion of genomes in IBD between source breed 1 and the breed with admixture and IBD_S2_ is the proportion of genomes in IBD between source breed 2 and the breed with admixture. The proportion of genomes in IBD is calculated as the proportion of admixed individuals’ genomes which are IBD with source breed 1 or 2. There are many algorithms to infer the state of IBD, and it is suggested to use the hidden Markov model to infer the tract of the IBD haplotype for each pair of individuals (BrianBrowning, 2011). The obtained rIBD scores are then used for mapping the introgressed genomic regions in the admixed breed. Our designed rIBD algorithm intends to identify these introgressed genomic regions in a pair-wise comparison between individuals so that an average rIBD score is calculated at a population level between the different breeds or populations.

The scale of the rIBD scores ranges, by definition, from −1 to 1. In an admixed breed, genomic regions with rIBD scores between −1 and 0 apparently have been introgressed more strongly from source breed 2, while genomic regions with rIBD scores between 0 and 1 apparently have been introgressed more strongly from source breed 1. Noting that with two breeds involved in an admixed population, the sum of IBD_S1_ and IBD_S2_ is 1, and an rIBD value of, for instance, 0.5 implies that this region has IBD_S1_ = 0.75 and IBD_S2_ = 0.25. Generally, the rIBD scores will follow a normal distribution, while genomic regions with extreme rIBD scores in the tails of the distribution indicate that these in the admixed breed are more similar to source breed 1 or source breed 2. It is also possible to calculate average rIBD scores from the sliding windows across the whole genome, which will reflect the genome-wide level of introgression between source breed 1 or 2 and the admixed breed. However, we focused more on the genomic regions showing clear signals with significant introgression from a certain source breed, that is, extreme positive rIBD scores or missing the rIBD scores from one side of the source breed, that is, extreme negative rIBD scores. Normally more efforts should be made to explore more in these genomic regions with a significant positive rIBD score or an extended genomic region with negative rIBD scores.

Finally, output options include outputting the rIBD scores in the sliding windows and plotting the rIBD scores across the chromosome. In this way, the user could save the rIBD scores calculated using the platform and also directly obtain an illustration of the rIBD scores across the chromosome. The users can use the sample data to demonstrate the output rIBD scores and the rIBD plot, and we used this as an example to show the results here.

## 3 Results and discussion

### 3.1 The relative identity-by-descent calculation platform

**FIGURE 1:**
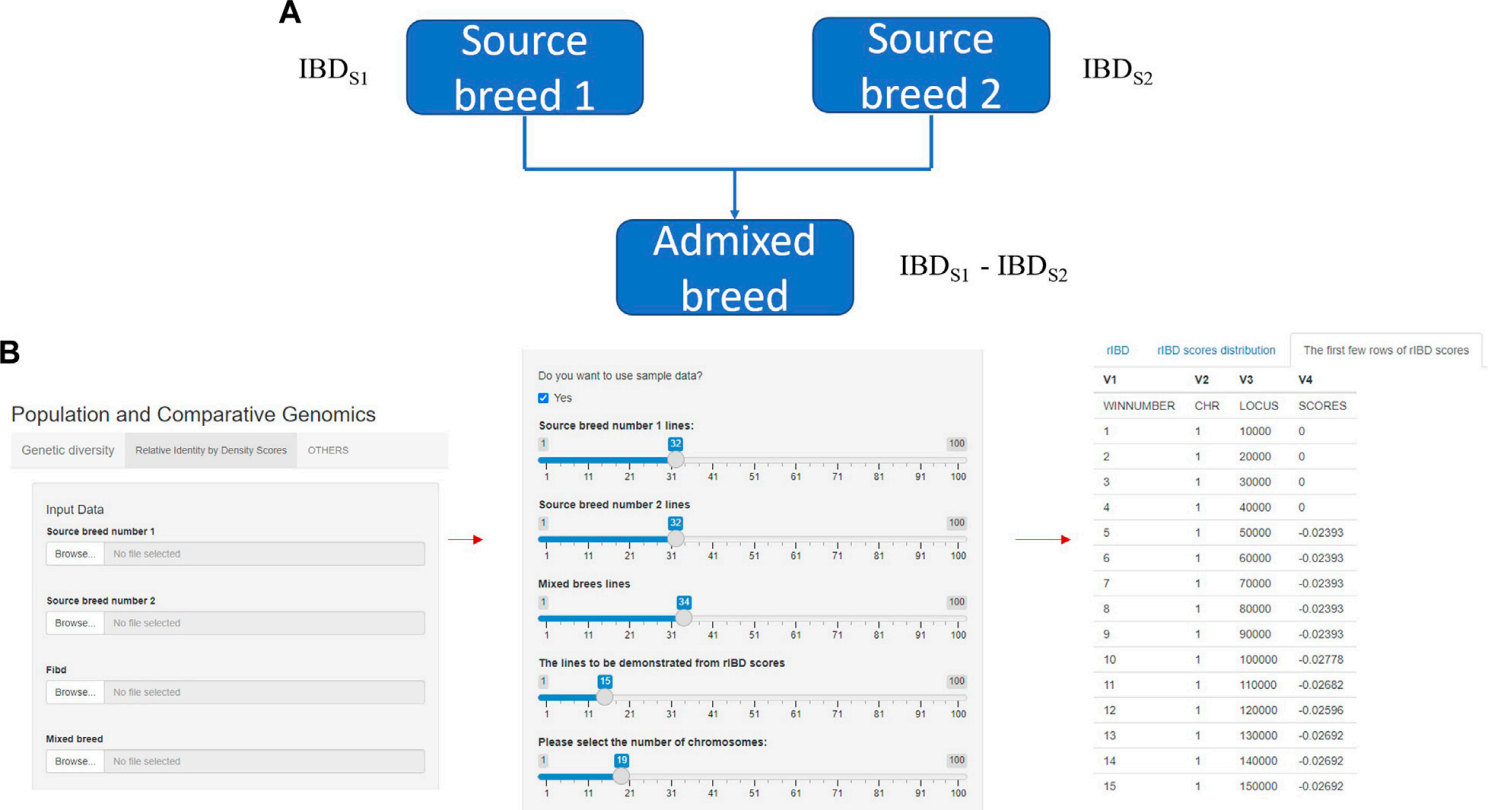
The core and main section of rIBD calculation platform and the process of calculating rIBD scores on the platform. **(A)** The core flow of the rIBD calculation algorithm; **(B)** The main section of rIBD calculation platform and the process of calculating rIBD scores on the platform.

rIBD algorithm is based on the relative difference of IBD on genomes between the source breed and the admixed breed (Figure 1A), and this algorithm is designed to identify genomic regions that have been subjected to adaptive introgression when the admixed breed has gene flow from two source breeds. We implement rIBD algorithm described in the Materials and methods section using the C programming language and Perl programming language and then re-write the programming part of the algorithm on the rIBD calculation platform. The rIBD calculation platform is available at https://cuibobetter.shinyapps.io/example-1/.

The rIBD platform is a user-friendly calculation platform with different options on the front interface. In the main section, the first step is to register as a user on our rIBD calculation platform. Then, the user should input the information from source breed 1, source breed 2, and an admixed breed and the basic IBD information in the main section (Figure 1A). The basic IBD information could be calculated using software packages such as Beagle (BrianBrowning, 2011). Usually, the breeds are clearly defined and the history of the breed is recorded, so it is possible to clearly define the source breeds and the admixed breed. The user also needs to specify the number of individuals from source breed 1, source breed 2, and the admixed breed and the number of chromosomes for this species (Figure 1B). After inputting these details in the main section, the rIBD calculation platform will output the rIBD scores for a sliding window of 10 kbp (Figure 1B). We only demonstrate the first few lines according to the user’s specification on the calculation platform so that the user has a direct illustration of the rIBD scores (Figure 1B). This output includes the chromosome number, the start and end positions of the corresponding sliding window in the size of 10 kbp, and the calculated rIBD scores in this window (Figure 1B). Meanwhile, it is also possible to utilize an executable of the rIBD calculation package for analyzing large amounts of data due to the limited space on the online platform as it is not possible to upload large-scale data on the platform, and the users should refer to the corresponding author of this work, for e.g., the executable of the rIBD algorithm.

Our rIBD calculation platform will be published as an online calculation platform at the end for the users around the world to calculate the rIBD scores and explore the genomic regions and the genetic basis of adaptive introgression. Our platform provides the users to automatically calculate either the rIBD scores from a specific genomic region or across the whole genome according to the capacity of our platform following the user’s requirement.

### 3.2 Analysis of data and illustrations of the calculated relative identity-by-descent scores on the platform

Here, we analyze a set of simulated genomic data from source breed 1, source breed 2, and one admixed breed. The aim of the analysis is to map the genomic regions that have been subjected to adaptive introgression in the admixed breed. Our rIBD platform is based on powerful Beagle, that is, the basic IBD states are first inferred from Beagle using HMM algorithm (BrianBrowning, 2011).

#### 3.2.1 Interpretation of output results

**FIGURE 2:**
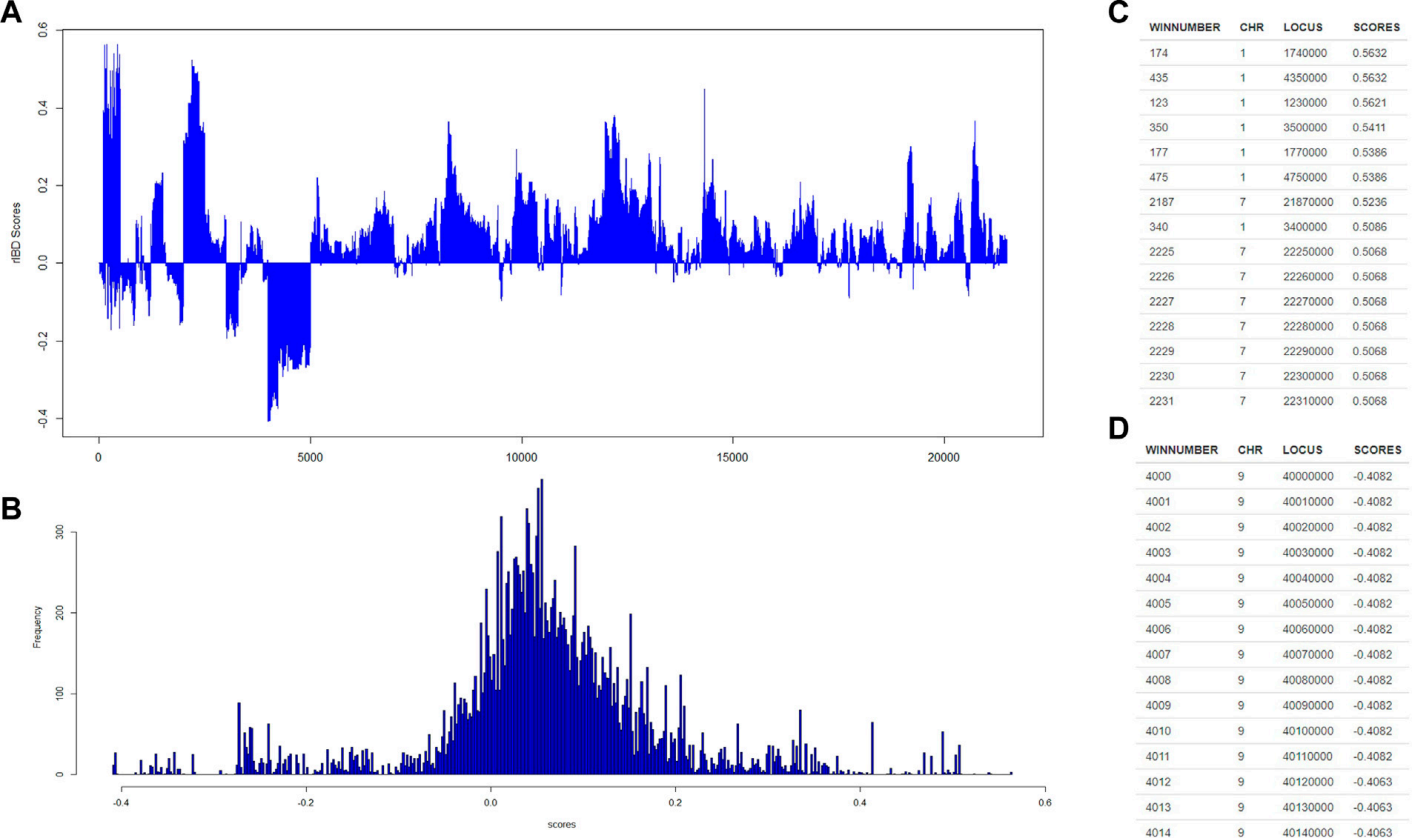
The distribution of rIBD scores across the chromosome and the significant positive or negative rIBD scores lists. **(A)** The calculated rIBD scores across the chromosome; **(B)** Tthe distribution of the rIBD scores; **(C)** The list of the significant positive rIBD scores list; **(D)** The list of the significant negative rIBD scores list.

Then, our rIBD platform is utilized to calculate the rIBD scores for this set of genomic data, and the rIBD scores are plotted across the whole genomic region (Figure 2A). The average rIBD score across this genomic region is 0.061, and the standard deviation of the rIBD scores is 0.124 (Figure 2B). This reflects that there is an overall introgression level of 0.061, which suggests that the introgression level from source breed 1 to the admixed breed is higher than that from source breed 2 to the admixed breed. The genomic regions with positive rIBD scores are more related with the introgression from source breed 1 to the admixed breed, which are the important genomic regions related with, for example, directional selection during the adaptive introgression process. However, the genomic regions with negative rIBD scores reflect the genes in the region with higher frequency due to introgression from source breed 2 to the admixed breed and directional selection later in the admixed breed during the adaptive introgression process.

#### 3.2.2 Identification of signals due to adaptive introgression with a high level of significance

Generally, the rIBD scores follow a normal distribution, and there are two extreme tails on the distribution (Figure 2B), showing that there are genomic regions with rIBD scores exceeding the significant level. We further take a very stringent cut-off of 0.432 and −0.310 calculated as three times the standard deviation from the mean in the normal distribution corresponding to 0.3% of the distribution for both positive and negative rIBD scores in order to identify the genomic regions with significant signals due to adaptive introgression. These significant genomic regions are mapped and listed as shown in Figures 2C,D. In this way, the genes in these genomic regions due to adaptive introgression can be mapped, and the gene list can be used to perform further functional studies.

**FIGURE 3:**
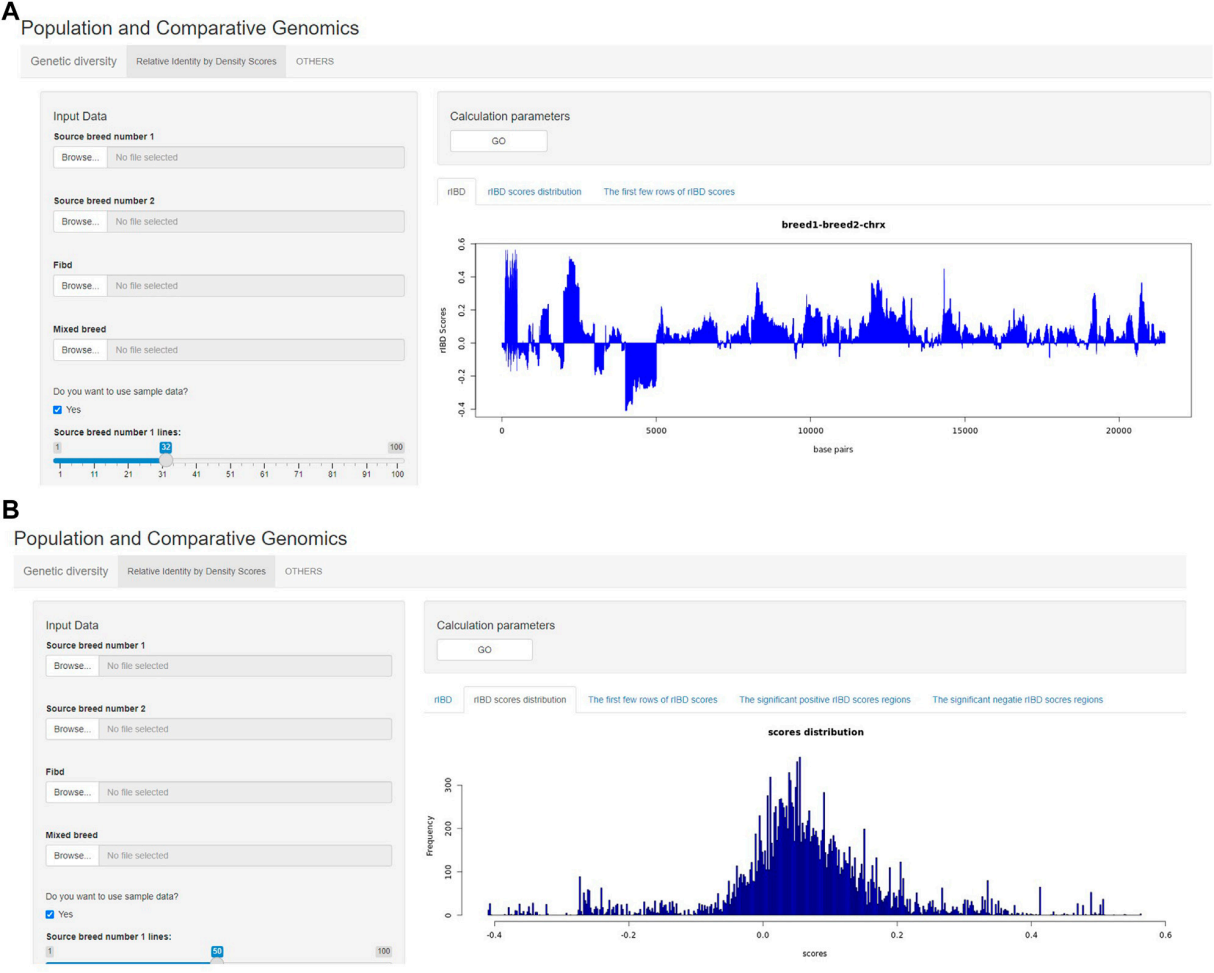
The demonstration of the output from the rIBD calculation platform. **(A)** The demonstration of the rIBD scores from the calculation platform; **(B)** The output rIBD distribution from the calculation platform.

The output of rIBD calculation can also be briefly demonstrated on the rIBD calculation platform. For example, the users could plot the rIBD scores across the genomic region to demonstrate the significant genomic regions with extreme high or low rIBD scores showing adaptive introgression signals from different source breeds (Figure 3A). Meanwhile, the users could also plot the distribution of the calculated rIBD scores to show the general pattern of the calculated rIBD scores and the extreme values of the calculated rIBD scores (Figure 3B). Generally, our rIBD calculation platform has compact functions and modules including inputting information, calculating rIBD scores, outputting the calculated rIBD scores, and illustrating the plots using the calculated rIBD scores for the users.

#### 3.2.3 Advantages of the relative identity-by-descent calculation platform

The advantage of our rIBD calculation platform is that the users are able to input the related information and calculate the rIBD scores directly without requiring any basic knowledge of bioinformatics. This would aid many agricultural breeders, especially from developing countries to obtain the introgression or long-term hybridization information between different breeds. We develop and implement a tool for the first time for rIBD score calculation integrated into a platform that can conveniently be used by agricultural breeders and researchers working in the conservation genetics area. It is necessary to mention that the amount of data which could be analyzed is limited due to the limited support to pay for the running of the standard online server at the moment. The amount of data to be analyzed and the ability of the calculations will also be increased and enlarged with the enlarging impact and usage of our rIBD calculation platform.

## 4 Conclusion

Exploring the genetic basis of long-term hybridization and adaptive introgression is extremely important for explaining evolutionary phenomena in biology, and a method with a corresponding tool to identify these genomic regions in detail during adaptive introgression is extremely useful for the researchers in the area. In this study, we developed the rIBD method and integrated into a calculation platform that is particularly useful for agricultural breeders and researchers working in the area of conservation breeding. Our rIBD method is based on pair-wise IBD comparisons between individual genomes from different source breeds and the admixed breed to map the specific genomic regions due to adaptive introgression. We then integrate the calculation platform with illustrations for the convenient utilization of users. We demonstrate the structure of our rIBD calculation platform and the usage of different modules of our rIBD calculation platform. Our rIBD calculation platform is a useful tool for the researchers working in conservation genetics and conservation breeding area and the agricultural breeders to study the genetic basis and map the genes in detail during adaptive introgression and long-term hybridization.

## Data Availability

Publicly available calculation platform is available and can be found at: https://39.106.33.52:3838/sample-apps/rIBD/. The executable version of the implemented rIBD algorithm can be required from the corresponding author when necessary.
