# Corrigendum: The computational implementation of a platform of relative identity-by-descent scores algorithm for introgressive mapping

**DOI:** 10.3389/fgene.2023.1296871

**Published:** 2023-11-03

**Authors:** Bo Cui, Zhongxu Guo, Hongbo Cao, Mario Calus, Qianqian Zhang

**Affiliations:** ^1^ School of Chemistry and Biological Engineering, University of Science and Technology, Beijing, China; ^2^ College of Water Resources and Civil Engineering, China Agricultural University, Beijing, China; ^3^ Institute of Biotechnology, Beijing Academy of Agricultural and Forestry Sciences, Beijing, China; ^4^ Department of Animal Science, Animal Breeding and Genetics Group, Wageningen University, Wageningen, Netherlands

**Keywords:** adaptive introgression, hybridization, identity by descent, gene flow, algorithm, computational platform

In the published article, there was an error in the Affiliations for Bo Cui, Zhongxu Guo, Hongbo Cao and Qianqian Zhang. Instead of “1. Institute of Biotechnology, Beijing Academy of Agricultural and Forestry Sciences, Beijing, China; 2. College of Water Resources and Civil Engineering, China Agricultural University, Beijing, China”, Bo Cui, Zhongxu Guo, and Hongbo Cao’s affiliations should be “1. School of Chemistry and Biological Engineering, University of Science and Technology, Beijing, China; 2. College of Water Resources and Civil Engineering, China Agricultural University, Beijing, China; 3. Institute of Biotechnology, Beijing Academy of Agricultural and Forestry Sciences, Beijing, China.” Instead of “1. Institute of Biotechnology, Beijing Academy of Agricultural and Forestry Sciences, Beijing, China”, Qianqian Zhang’s affiliations should be “1. School of Chemistry and Biological Engineering, University of Science and Technology, Beijing, China; 3. Institute of Biotechnology, Beijing Academy of Agricultural and Forestry Sciences, Beijing, China.”

The authors apologize for this error and state that this does not change the scientific conclusions of the article in any way. The original article has been updated.

